# Metabolomics and Microbiomes as Potential Tools to Evaluate the Effects of the Mediterranean Diet

**DOI:** 10.3390/nu11010207

**Published:** 2019-01-21

**Authors:** Qi Jin, Alicen Black, Stefanos N. Kales, Dhiraj Vattem, Miguel Ruiz-Canela, Mercedes Sotos-Prieto

**Affiliations:** 1 Division of Food Sciences and Nutrition, School of Applied Health Sciences and Wellness, Ohio University, Athens, OH 45701, USA; qj196613@gmail.com (Q.J.); ab914017@ohio.edu (A.B.); vattem@ohio.edu (D.V.); 2 Department of Environmental Health, Harvard T.H Chan School of Public Health, 677 Huntington Avenue, Boston, MA 02115, USA; skales@hsph.harvard.edu; 3 Edison Biotechnology Institute, Ohio University, Athens, OH 45701, USA; 4 Department of Preventive Medicine and Public Health, University of Navarra, 31008 Pamplona, Spain; mcanela@unav.es; 5 IDISNA, Navarra Health Research Institute, 31008 Pamplona, Spain; 6 CIBER Fisiopatología de la Obesidad y Nutrición (CIBER Obn), Instituto de Salud Carlos III, 28029 Madrid, Spain; 7 Diabetes Institute, Ohio University, Athens, OH 45701, USA

**Keywords:** Mediterranean diet, metabolomics, microbiome

## Abstract

The approach to studying diet–health relationships has progressively shifted from individual dietary components to overall dietary patterns that affect the interaction and balance of low-molecular-weight metabolites (metabolome) and host-enteric microbial ecology (microbiome). Even though the Mediterranean diet (MedDiet) has been recognized as a powerful strategy to improve health, the accurate assessment of exposure to the MedDiet has been a major challenge in epidemiological and clinical studies. Interestingly, while the effects of individual dietary components on the metabolome have been described, studies investigating metabolomic profiles in response to overall dietary patterns (including the MedDiet), although limited, have been gaining attention. Similarly, the beneficial effects of the MedDiet on cardiometabolic outcomes may be mediated through gut microbial changes. Accumulating evidence linking food ingestion and enteric microbiome alterations merits the evaluation of the microbiome-mediated effects of the MedDiet on metabolic pathways implicated in disease. In this narrative review, we aimed to summarize the current evidence from observational and clinical trials involving the MedDiet by (1) assessing changes in the metabolome and microbiome for the measurement of diet pattern adherence and (2) assessing health outcomes related to the MedDiet through alterations to human metabolomics and/or the microbiome.

## 1. Introduction

The Mediterranean diet (MedDiet) is a dietary pattern that emphasizes the intake of vegetables, fruits, nuts, whole grains, fish, and unsaturated fats and vegetable oils (mainly olive oil) and aims to limit the intake of butter, sweets, and red and processed meat [1,2]. Multiple studies have found evidence of lower risk and incidence of and mortality from chronic metabolic diseases with adherence to the MedDiet [3,4,5,6]. The United States (US) Department of Agriculture and Health and Human Services recognized the MedDiet as a healthy dietary pattern in the 2015–2020 Dietary Guidelines for Americans [7,8], and this dietary pattern has been successfully adapted into workplace interventions [9]. Notwithstanding the MedDiet’s growing acceptance for health improvement, the accurate assessments of exposure and adherence to the MedDiet in epidemiological studies and clinical interventions, respectively, continue to be major hurdles in advancing our understanding of the MedDiet’s efficacy. Additionally, the underlining molecular and metabolic mechanisms that influence health outcomes in response to the MedDiet have not been elucidated.

The inherent subjectivity in the self-administered instruments used for dietary adherence/compliance assessment, such as food frequency questionnaires, multiple-day food records, and 24-h dietary recall, creates susceptibility to the introduction of bias and errors into data collection, ultimately impacting study outcomes [10,11,12]. There is an increasing consensus that employing objective measurement approaches (e.g., biomarkers and metabolic signatures) and robust research design (controlling for regression to the mean) concurrently with self-administered instruments may significantly improve data quality and validate study outcomes. However, logistical challenges, the economic burden, and the absence of reliable and sensitive biomarkers for dietary patterns, as opposed to an individual nutrient/ingredient, have limited the inclusion of objective analytical approaches in study methodologies. In this regard, leveraging rapid advances in high-throughput, big data omics technologies may overcome such pitfalls in nutritional epidemiology and intervention studies. Specifically, metabolomics (the study of low-molecular-weight metabolites in biological samples) and the research of microbiomes (the study of host-enteric microbial ecology) may facilitate the discovery and validation of specific biomarker signatures of dietary behavior and probe the complex interactions between food, lifestyle, and disease [13,14]. Generally, the most commonly used techniques in metabolomics analysis are mass spectrometry (MS), usually coupled with gas or liquid chromatography, and nuclear magnetic resonance (NMR) spectroscopy [15]. Each of these techniques has its own advantages and disadvantages. Both can be used to identify the structure of metabolites and measure the absolute and relative concentrations of metabolites. However, NMR has higher reproducibility and is more reliable for determining concentrations, while MS is more sensitive [15,16]. As for the analysis of the microbiome, techniques have evolved from culture-dependent methodologies to novel, next generation sequencing tools. The latter has allowed for the taxonomic and phylogenetic evaluation of the bacterial community based on 16S RNA gene amplicon analysis [17].

There is a growing body of knowledge on the metabolic signatures of individual food groups, such as fruit (proline betaine as a biomarker of citrus fruits) [18,19,20,21,22], vegetables (S-methyl-L-cysteine sulphoxide and its derivatives for cruciferous vegetables) [23,24,25,26], and whole grains (urinary excretions of alkylresorcinols and benzoxazinoid) [23,27,28,29] (Supplemental Table S1). However, notwithstanding the scientific interest in the matter, the literature on the metabolomics of overall diet patterns (including the MedDiet) is limited [30].

The importance of the gut microbiome in food digestion, nutrient metabolism, micronutrient production, enteric immunity, and gut function have been known for a long time [31,32]. Seminal papers published in the past two decades have expanded the understanding of the gut microbiome to overall human health and exposed the multilevel dimensional complexity of the diet–environment–microbiome–host interactions that impact human health [33,34,35]. The rapid expansion in this research area, in this regard, has disrupted the functional locality of the microbiome and highlighted its systemic effects beyond the gut, which are mediated by a combination of direct (physical) interactions of the microbes with the host and indirect processes via the production of metabolites, de novo or from diet. Moreover, the plasticity of the microbial ecology in response to diet and the growing evidence of the association between an altered ecological profile of the microbiome (dysbiosis) and disease have positioned the targeted and directed modulation of the gut microbiome as an exciting therapeutic target for a wide variety of pathologies, including acute infections (*Clostridium difficle*) [36], chronic illnesses (metabolic disease) [37], and mental health [38].

Sustained scientific exploration of the direct manipulation of gut ecology has identified strain-level specificity of microorganisms (e.g., lactic acid bacteria) for different health indications, as well as diet-derived natural or synthetic compounds that can stimulate colonization (fructooligosaccharides) and delivery mechanisms (encapsulation) to ensure survival and viability during gastrointestinal transit. The relative simplicity of this approach, favorable consumer perception, and historical correlation of fermented diets with health has allowed for the successful commercial exploitation of these principles by the food and beverage industry on a global scale. The rapid proliferation of consumer products (probiotic/prebiotics) and the dramatic growth of the size of this market continue to raise new instances of regulatory scrutiny and topics of policy debate [39].

The body of knowledge on the ‘indirect’ interactions between diet, the microbiome, and health via metabolite production is, on the other hand, relatively nascent [40]. The chemical complexity of diet, gut microbial diversity, and host-related genetic and lifestyle factors have been hurdles that have impeded investigations that attempt to reliably study even just the interaction of an individual dietary ingredient with health. Moreover, these challenges are amplified when attempting to delineate the effects of dietary patterns (e.g., the MedDiet) as opposed to one specific dietary ingredient (e.g., polyunsaturated fatty acid (PUFA)) on health outcomes. These problems are compounded when a potential metabolite of interest is produced both in the host and by the microbiome. For example, levels of trimethylamine oxide-N-oxide (TMAO) and its precursors choline and carnitine have been correlated with cardiovascular outcomes. However, establishing the relative contributions of the host, diet, and microbial dysbiosis have proven to be challenging in nutrition studies.

The application of big data and high-throughput omics approaches have successfully transformed physical, medical, forensic, and environmental sciences [41,42,43]. The combination of metabolomics and the study of the microbiome using powerful big data statistics can be leveraged to improve our understanding of the relationship between the MedDiet and health. In this narrative review, we summarize current scientific evidence from epidemiological studies focused on (1) the assessment of changes in the metabolome and microbiome for the measurement of adherence to the MedDiet pattern and (2) the understanding of the effect of the MedDiet on health outcomes through alterations in human metabolomics and/or the microbiome.

## 2. Metabolomics and the Mediterranean Diet—The Present Status

Strong evidence supports the use of the MedDiet as a preventive strategy to lower the risk of cardiovascular diseases (CVD) [44,45,46]. However, the characterization of the dietary pattern has been hindered due to the complex interactions between the human genome and diet [30,45]. Recent research in nutritional metabolomics has focused on discovering new biomarkers of nutritional exposure, nutritional status, and nutritional impacts on disease [47]. There is general consensus that establishing reliable biomarkers is imperative to facilitate the development of “precision nutrition”, in which omics techniques can be applied to a personalized diet for the prevention and management of disease. Although relevant scientific literature is emerging on this topic, with several studies pursuing the identification of metabolic signatures for overall dietary patterns, few have focused specifically on the MedDiet [30]. Moreover, a collective endeavor in this regard can also be extended to discover biomarkers of health outcomes following a MedDiet intervention or dietary approaches [48]. Along these lines, this section will summarize the current status of the research on using metabolomics to assess the MedDiet pattern and to understand the associations between the MedDiet and health.

### 2.1. Metabolomics Approach as an Assessment of Adherence to the Mediterranean Diet

A recent review of the use of nutritional metabolomics to assess dietary intake found 16 studies on dietary patterns and metabolomics, of which only three specifically assessed the MedDiet pattern [30]. Here, we include three additional articles regarding metabolomics and dietary patterns in general [49,50] and four specifically about the MedDiet [51,52,53]. Metabolomics profiles have been characterized in multiple dietary patterns, including the New Nordic diet, low-fat diet, low glycemic index diet, very-low-carbohydrate diet, a diet concordant with World Health Organization (WHO) healthy eating guidelines, prudent diet, etc. A recent study assessing overall dietary patterns was a randomized, controlled, crossover study that used a targeted metabolomics method to assess concentrations of 333 plasma metabolites of three hypocaloric dietary patterns—low-fat, low glycemic index, and very-low-carbohydrate diets—to evaluate compliance with each for a 4-week period [49]. Using four types of constructed, Bayesian network classifiers, the plasma metabolites that were different from at least one other diet were diacylglycerols (DAG), triacylglycerols (TAG), and branched-chain amino acids (BCAA) [49]. For example, the low glycemic index diet had an intermediate concentration of most metabolites (105 out of 152), which differentiated it from the other two diets [49]. These metabolites included hippurate 5-aminolevulinic acid, pipecolic acid, cytosine, triacylglycerides, and hydroxyproline [49]. Another study identified two distinct dietary patterns using dietary and urinary metabolomics data obtained from the National Adult Nutrition Survey [50]. They used two-step cluster analysis in both urine and dietary data and identified in the latter a “healthy” and an “unhealthy” cluster [50]. Consistent with the previous studies, the authors found higher levels of hippurate and also betaine, anserine, N-phenylacetylglutamine, 3-hydroxybutyrate, citrate, tryptophan, and 2-aminoadipate in the healthy cluster [50]. Higher creatinine, glycylproline, N-aceytalglutamate, and theophylline were found in the unhealthy cluster, and the two dietary patterns were further supported by subsequent validation in the NutriTech food intake study [50]. Recently, the serum metabolic profile of the Dietary Approaches to Stop Hypertension (DASH) pattern identified the top 10 most influential known metabolites, which differed significantly between participants randomly assigned to the DASH diet compared with both the control diet and the fruit and vegetables diet [54].

With regards to the MedDiet pattern, while still emerging, the evidence about metabolomics and the MedDiet–health association remains limited [48]. Compared with general dietary patterns, the number of published studies exploring the metabolomics signatures of the MedDiet is relatively small. In addition to those studies addressed by Guasch et al. [30], we describe here the evidence of four additional studies. Table 1 provides a more detailed summary of the evidence from studies using metabolomics to assess potential biomarkers for the MedDiet. González-Guardia and colleagues investigated biomarkers in a crossover study with four isocaloric diets: the MedDiet supplemented with Coenzyme Q_10_ (Med + CoQ), the MedDiet, the Western diet high in saturated fat, and a low-fat, high-carbohydrate diet rich in n-3 polyunsaturated fat [55]. Greater urinary hippurate levels after the adherence to the Med + CoQ diet and greater phenylacetylglycine levels after adherence to the Western diet high in saturated fat were found in female participants [55]. This higher level of hippurate was reported to positively correlate with CoQ and plasma β-carotene levels and negatively correlate with gene expression of transcription factor Nrf2, thioredoxin, superoxide dismutase 1, and the gp91phox subunit of NADPH (nicotinamide adenine dinucleotide phosphate) oxidase gene expression [55].

Another cross-sectional study, using a targeted metabolomics approach explored the association between the effect of the interaction between polymorphisms and the MedDiet on the levels of 14 specific serum metabolites that could be related to dietary adherence [56]. These metabolites played key roles in gene-encoding enzymes modulated by nine previously genotyped polymorphisms [57,58,59]. The results showed that a 1 unit increase in adherence to the MedDiet pattern was associated with a 5% increase in 5-Methylfolate (5-MTHF) [56]. Finally, another cross-sectional study found that urine microbial metabolites (phenylacetylglutamine, p-cresol, and 4-hydroxyphenylacetate) defined high adherence to the MedDiet [60].

### 2.2. Metabolomics, the Mediterranean Diet, and the Association with Health

Several studies have investigated the role of metabolites on the etiological and patho-physiological processes implicated in cardiovascular disease and related morbidities. For example, in a meta-analysis, the results from prospective studies support robust positive associations of BCAAs (leucine, isoleucine, and valine) and aromatic amino acids (tyrosine and phenylalanine) and inverse associations of glycine and glutamine with incident type 2 diabetes (T2D) [62]. Similarly, the results from a systematic review showed that the metabolites associated with CVD risk were acylcarnitines and dicarboxylacylcarnitines, TMAO, and several amino acids, such as phenylalanine, glutamate, and several lipid classes [63]. Although it is known that the MedDiet is associated with lower risk of CVD, attempts to correlate MedDiet-adherence-derived metabolites with CVD-associated biomarkers and clinical outcomes have proved inconclusive. For example in the randomized, controlled OmniHeart study, blood pressure was found to be significantly associated with six urinary metabolites reflecting dietary intake [64], and among those following the New Nordic Diet, higher levels of vaccenic acid and 3-hydroxybutanoic acid were related to higher weight loss, while higher concentrations of salicylic, lactic, and N-aspartic acids and 1,5-anhydro-d-sorbitol were related to lower weight loss [65]. Evidence from studies specifically addressing the MedDiet come from the PREDIMED (Prevención con Dieta Mediterránea) study (a randomized, controlled trial with a 4.8-year follow-up) that has focused on baseline and 1-year changes in metabolites after following the MedDiet supplemented with extra virgin olive oil (EVOO) or nuts in comparison with a low-fat diet and its association with CVD and T2D. In this section, we summarized the evidence of metabolomics relating to the MedDiet pattern and its association with CVD and T2D (Table 2).

**Figure 1:**
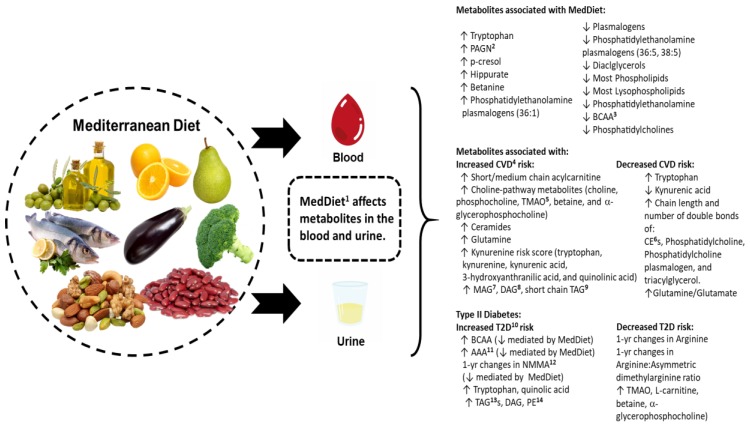
Depiction of the metabolites associated with adherence to the MedDiet in the absence and presence of disease. All the increases in the metabolite profiles are identified with an up arrow, and all the decreases are identified with a down arrow. In general, the results from the PREDIMED study suggest that the MedDiet may counteract the deleterious effect of metabolites related to CVD and diabetes and that it is able to reduce the levels of some of them (e.g., tryptophan and BCAA) after one-year of intervention in comparison with the control group. ^1^ MedDiet: Mediterranean diet. ^2^ PAGN: phenylacetylglutamine. ^3^ BCAA: branched-chain amino acids. ^4^ CVD: cardiovascular disease. ^5^ TMAO: Trimethylamine N-oxide. ^6^ CE: cholesterol ester. ^7^ MAG: monoacylglycerols. ^8^ DAG: diacylglycerols. ^9^ TAG: Triacylglycerol. ^10^ T2D: type 2 diabetes. ^11^ AAA: aromatic amino acid. ^12^ NMMA: N-methylmalonamic acid. ^13^ TAG: triacylglycerols; ^14^ PE: phosphatidylethanolamines.↓: decreased with the consumption of MedDiet. ↑: increased with consumption of MedDiet.

All the studies evaluating the metabolites in the PREDIMED study followed a nested case–cohort study design, by which between 10–20% of the original study participants were randomly selected along with all the incident cases of CVD (*n =* 229–233) or diabetes (*n =* 251). The studies focusing on CVD, were assessing most of the metabolites already identified to be associated with CVD, including the following: BCAAs, choline pathway, different classes of lipids, ceramides, tryptophan, acylcarnitines, and glutamine. Higher concentrations of short and medium acylcarnitines [66], choline pathway metabolites (TMAO, betaine, choline, phosphocholine, and α-glycerophosphocholine) [67], kynurenine risk score [68], ceramides [69], glutamine [70], and some lipids (monoacylglicreol (MAG), DAG, short-chain triacylglycerol (TAG)) [71] were associated with increased CVD risk at the baseline. On the contrary, higher tryptophan, cholesterol esters (CE), glutamine/glutamate ratio, and polyunsaturated phosphatidylcholines were associated with lower risk of CVD (Figure 1). When the one-year changes in the metabolites were assessed, their associations with CVD were less clear. Only changes in acylcarnitynes [66] and phosphatidylethanolamine [71] were associated with the composite of CVD risk. Similarly, the role of the MedDiet in mediating the association seemed to be more significant when baseline metabolites were assessed. The MedDiet counteracted the deleterious effect of the high tryptophan risk score [68] and modulated the risk at the baseline for acylcarnitines [66], choline [67], ceramides [69], and glutamine [70], demonstrating the cardioprotective effects of the MedDiet by shedding some light on the biological mechanisms.

As with CVD, some metabolites have been associated with T2D (nested case–cohort PREDIMED study with around 250–251 cases of T2D) (Table 2). Higher concentrations of BCAAs, aromatic amino acid (phenylalanine and tyrosine) [72], changes in ornitin and citrulline [73], triacylglycerols (TAG), DAG, phosphatidylethanolamines (PE) [74], tryptophan, and one-year changes in quinolic [75] were associated with higher risk of T2D. In contrast, TMAO, L-carnitine, betaine, α-glycerophosphocholine [76], and arginine: asymmetric dimethylarginine (ADMA) [73] were associated with lower risk of T2D. Only BCAAs, aromatic amino acid, and tryptophan interacted with the MedDiet + EVOO intervention to modulate the risk of T2D [72] (Figure 1).

In summary, the above studies demonstrated extensive variability in the metabolomic signatures of the MedDiet pattern, disease biomarkers, and clinical health outcomes (Figure 1). These may result from disparities in the biological samples (urine versus plasma/serum), the frequency of metabolite analysis, the analytical methods/techniques, inter-laboratory variability, and differences in the study design, the participant health status, and the MedDiet composition (e.g., Mediterranean countries have a higher consumption of olive oil than non-Mediterranean countries). Furthermore, different self-reported dietary assessment tools and inherent recall errors and biases exacerbate metabolomic profile inconsistencies across different MedDiet studies. Future approaches should attempt to standardize the above stated methodological discrepancies and also validate the findings in wider populations, especially for the MedDiet, for which little information is available outside of the PREDIMED study.

## 3. Microbiome and the Mediterranean Diet—The Present Status

The human gut is a highly diverse ecosystem consisting of 10–100 trillion microbial cells that are mostly bacteria, but it also includes viruses, archaea, fungi, and others [46,80]. Quantitatively, the total number of microbial genes in the gut or ‘gut microbiome’ outnumbers the host (human) genome by 100 to 1 [81], and they may significantly impact the host’s physiology and ultimately health. Recent advances in 16S rRNA-based sequencing technologies have facilitated species-level identification of several of these non-culturable bacteria, with the majority belonging to the phyla Firmicutes, Bacteroidetes, Actinobacteria, and Proteobacteria [46,82,83]. Evidence suggests that there are complex and dynamic associations between the host’s health, diet, lifestyle, and environment and the composition of the host’s gut microbiome throughout the host’s lifespan [84]. Elucidating causal relationships between intrinsic/extrinsic host factors on the gut microbiome and the impact of the gut microbiome on metabolic functions, immunity, and health outcomes is currently an area of active investigation globally [85,86,87,88,89,90].

It has been estimated that about 57% of the microbiome’s entire variation is due to dietary habits, emphasizing the importance of diet in gut microbial ecology [91]. However, studies exploring the relationship between the gut microbiome and diet have primarily focused on single nutrients or foods and microbial response [92]. The literature describing the influence of dietary patterns, a more comprehensive determinant of health, on the microbiome is very limited. For example, with respect to the MedDiet, several studies have assessed the impact of olive oil [93], red wine [94], whole grains [95], or other individual components on microbiota, but few have analyzed the MedDiet pattern in its entirety [92]. Research on the MedDiet’s effects on the composition of the microbiome and the contribution of the composition of the microbiome to MedDiet-related health outcomes will be reviewed in the next two sections.

### 3.1. Mediterranean Diet Effects on General Microbiome Composition

Although microbiome changes in response to habitual dietary intake are not very well understood, general associations between some dietary components/patterns and microbiota have been noted in the literature. For example, elevated levels of *Bacteroides sp*. in the gut have been observed with a Western-style dietary pattern that is rich in animal protein and saturated fat, while gut *Prevotella sp*. have been shown to increase with the consumption of carbohydrates, especially from vegetables and grains, or a more Mediterranean-style diet [46,96,97]. As described above, the physiological effects of the microbiome are mediated by a combination of direct (physical) interactions of the microbe with the host and indirect processes via the production of fermentation metabolites, *de novo* or from diet. The profile of gut bacterial metabolites from fermentation is a function of both the bacterial ecology and the substrate (i.e., fermentation of dietary fiber and other complex carbohydrates, which are prominent in the MedDiet, or other plant-based foods results in short-chain fatty acids (SCFA) as opposed to TMAO, which is usually seen in populations consuming diets rich in red meats). Table 3 lists studies that evaluated the consumption of the MedDiet and its effects on the microbial composition in healthy individuals. A recent, comparative, cross-sectional study explored the differences between two groups of teenagers from different geographical locations (Egypt versus United States) with respect to their microbial ecology, enterotype clustering function, and metabolite production in response to their dietary intake. The authors found that in the group of Egyptian teenagers, who were primarily exposed to a Mediterranean-style diet, all the gut microbial communities belonged to the *Prevotella* enterotype. In contrast, almost all of the American teenagers in the study had gut microbial communities belonging to the *Bacteroides* enterotype [96]. In an earlier 10-day controlled-feeding study that examined gut microbial enterotypes as a result of high-fat/low-fiber or low-fat/high-fiber diets, Wu et al. found similar results but additionally observed that the enterotype of the host microbiome remained unaltered as the relative distributions (abundance) of bacterial phyla and genera levels changed within 24 h of initiating a new diet pattern [97]. However, the magnitude of the changes was modest and was not sufficient to switch the individuals between the enterotype clusters associated with protein/fat and carbohydrates, suggesting an association between a long-term Mediterranean-style diet and enterotype partitioning [97].

In a randomized controlled trial, Djuric and colleagues explored the relationship between carotenoid concentrations and mucosal colonic bacteria in healthy individuals at an increased risk of colon cancer. Colonic biopsy samples (*n =* 88 at baseline, *n =* 82 after dietary intervention) were obtained from the Healthy Eating study, in which the participants were assigned to one of two dietary interventions, a MedDiet or a Healthy Eating diet for 6 months, with the primary difference being the total fat content of the diet [98,99]. The researchers found that after the interventions, the colonic mucosal bacteria was not significantly altered. However, 11 operational taxonomic units were significantly associated with increased serum carotenoid concentrations, including lower Firmicutes taxa and higher Proteobacteria abundance. Additionally, an increased abundance of *Prevotella sp*. was observed in the highest carotenoid tertile [100]. These findings are consistent with other “short-term” dietary intervention studies on the microbiome and suggest that long-term dietary patterns may have a greater impact on changes in microbial ecology. The results from other cross-sectional studies also evidence the higher abundance of *Prevotella sp*. with greater adherence to the MedDiet [92,101]. De Filippis et al. found that *Prevotella sp*. was specifically associated with fiber-degrading Firmicutes and increased levels of fecal SCFA, which has been associated with health benefits [40]. Consistent with the previous section, higher urinary TMAO was associated with lower adherence to the MedDiet. Another cross-sectional study assessing the association between the MedDiet and microbial-derived phenolic compounds in stool samples (*n =* 74) found that subjects with greater MedDiet scores had statistically higher levels of *Clostridium* cluster XIVa and *Faecalibacterium prausnitzii*, which is a butyrate producer with proposed anti-inflammatory properties. This suggests the MedDiet may produce more beneficial bacteria in the gut, which produces healthy metabolites. They also found that *Akkermansia sp*., Bacteroides*-Prevotella-Porphirimonas sp*., and *Bifidobacterium sp*. trended higher in the 42 subjects scoring ≥4 on the MedDiet score (range 0–8); however, there was a smaller presence of the *Lactobacillus sp*. group in the greater MedDiet adherence group [102].

In an earlier cross-sectional study, Gutierrez-Diaz and colleagues explored the effects of the MedDiet in 31 healthy subjects. The study found that several individual components of the MedDiet were associated with an abundance of specific gut taxa. For example, cereals were linked to *Bifidobacterium sp*. and *Faecalibacterium sp*., olive oil with Tenericutes and *Dorea sp*., red wine with *Faecalibacterium sp*., vegetables with Rikenellaceae, *Dorea sp*., *Alistipes sp.*, and *Ruminococcus sp*., and legumes with *Coprococcus sp*. The study also observed direct associations between the MedDiet classifications and the abundance of the phylum Bacteroidetes and family Prevotellacceae. Additionally, the genus *Prevotella sp*. was found to be inversely related to the phylum Firmicutes and the genus *Ruminococcus sp*. [92]. This lower ratio of Firmicutes–Bacteroides with higher adherence to the MedDiet was also shown in a recent study with 27 healthy individuals [103]. In agreement with the previous research, higher concentrations of fecal propionate and butyrate were detected, corroborating the potential mediated health effects of the MedDiet by SCFA production.

In summary, the MedDiet affects the composition of the gut microbiota (such as higher *Prevotella sp*., *Clostridium* cluster XIVa, *F*. *Pprausnitzii*, Bacteroides, and *Bifidobaterium sp*. or lower Firmicutes and Bacteroides) and can affect the functionality, diversity, and activity of some bacteria, whose metabolites can have health benefits (such as SFCA). Further studies evaluating temporal changes to the colonization, stability, and enterotype changes of the gut microbiota in clinical studies are still warranted to establish a gut microbiota composition as a marker of MedDiet adherence.

### 3.2. Microbiome, the Mediterranean Diet, and the Association with Health

While extensive research exists in the literature regarding the health benefits of following a MedDiet pattern, there is limited knowledge regarding the MedDiet’s impact on microbiome-mediated disease outcomes. In this section, the literature on microbiome changes as they relate to MedDiet consumption in individuals with known diseases and the contribution of microbiome changes to disease risk will be reviewed and summarized in Table 4.

Emerging evidence is showing that the adherence to a MedDiet is associated with more gut microbial diversity. In the CORonary Diet Intervention with Olive Oil and Cardiovascular PREVention (CORDIOPREV) study, 138 participants with metabolic syndrome (MetS) and 101 without MetS were randomized into two groups. For 2 years, both groups underwent either a MedDiet or a low-fat diet intervention. At basal time, a higher abundance of *Bacteroides*, *Eubacterium* and *Lactobacillus* generas were found while *Bacteroides fragilis* group, *Parabacteroides*. *distasonis*, *Bacteroides thetaiotaomicron*, *Faecalibacterium prausnitzii*, *Fusobacterium*. *nucleatum*, *Bifidobacterium*. *longum*, *Bifidobacterium adolescentis*, *Ruminococcus flavefaciens* subgroupm and *Eubacterium rectale* were decreased in the participants with MetS when compared to those without. After the two-year intervention, MedDiet adherence increased the abundance of *P*. *distasonis*, *B*. *thetaiotaomicron*, *F*. *prausnitzii*, *B*. *adolescentis*, and *B*. *longum* in the MetS patients, suggesting that a long-term MedDiet partially restored the dysbiosis in the gut microbiota in the MetS patients, although the MetS persisted.

In another study conducted by the same research team, the effects of both a MedDiet and a low-fat diet were examined but this time on the restoration of the microbiome in three different groups of participants. A total of 106 subjects were evaluated: 33 were obese with MetS, 32 were obese without MetS, and 41 were not obese and did not have MetS. The researchers found that the MedDiet and low-fat diet consumption restored the microbiota in the MetS–obese participants to the gut microbiome found in metabolically healthy people. However, no significant changes in gut microbial composition occurred if MetS was absent (non-MetS–non-obese or non-MetS–Obese). Specifically, the study showed that the Firmicutes/Bacteroidetes ratio (which has been linked to obesity) was related to the presence or absence of metabolic traits and not with obesity itself. The Met–Obese group, previously described with a reduction of genera with saccharolytic activity, showed increases in *Bacteroides*, *Prevotella* (both forming bacteroidetes phylum), *Faecalibacterium*, *Roseburia*, *Ruminococcus* generas, and *P*. *distasonis* and *F*. *prausnitzii*, a decreasing Firmicutes/ Bacteroidetes ratio, and no effect on the abundance of Streptococcus or Clostridium. This is important because the restoration of saccharolytic activity genera are associated with an increase in fermentation capacity to SCFA, for which health benefits have previously been described. Consistent with those results, an increased abundance of *Roseburia sp*. and *Oscillospira sp*. and insulin sensitivity were found in 20 obese subject after following a one-year MedDiet intervention [104]. This butyrate-producing genus (*Roseburia)* has been found to have anti-inflammatory effects and has a lower abundance in T2D [105,106]. Interestingly, *F*. *prausnitzii*, which in the previous literature had been significantly increased with MedDiet consumption [102,107], increased significantly only in the low-fat diet group. This study also showed that some changes in fecal and plasma metabolites could also be linked to changes in gut microbiota [102]. Overall, the results of this study suggested that a MedDiet could be used to prevent and manage T2D, although more research is necessary to associate these therapeutic effects with microbiome-related factors [104].

Other studies exploring the MedDiet’s influence on inflammation associated with Crohn’s disease (CD) found that 6 weeks of MedDiet intervention had no significant effect on the microbial ecology relative to the control group. However, a trend towards “normal” composition (Bacteroidetes and *Clostridium* clusters IV and XIVa increased, and the abundance of Proteobacteria decreased as did Bacillaceae) was found in this study. Notwithstanding the lower number of subjects (*n =* 8), shorter duration, and absence of a significant alteration in the microbial ecology in this particular study, it should be noted that study participants were already under dietary management of CD, which may have altered their microbiota before the study [83]. Finally, Mitsou et al. explored the relationship between MedDiet adherence with gut microbiota composition and gastrointestinal (GI) symptomology. A profiling analysis showed that participants with high adherence to the MedDiet had significantly less *Escherichia coli*, a higher ratio of *Bifidobacteria to E. Coli*, and increased levels of *Candida albicans* and SCFA [108]. Interestingly, the MedDiet was also correlated with the alleviation of undesirable GI symptoms and fecal moisture.

**Figure 2:**
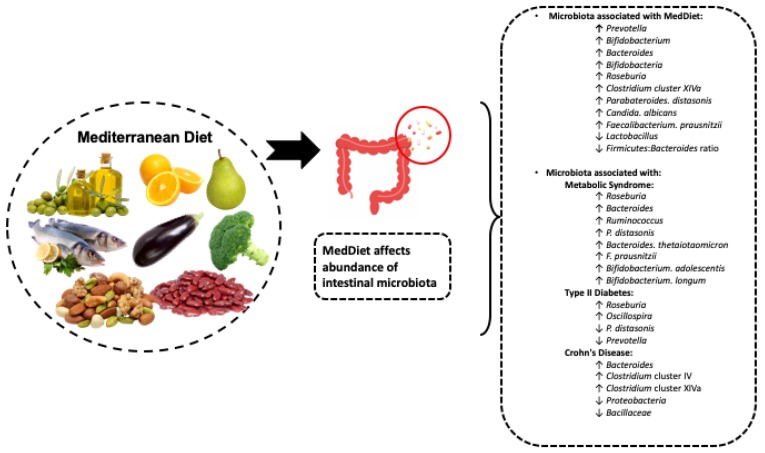
Depiction of the microbiota associated with MedDiet consumption in the absence and presence of disease. All the increases in the microbial changes are identified with an up arrow, and all the decreases are identified with a down arrow. General alterations in the microbiota were only indicated if two or more studies identified that same bacterial change. Research on microbial responses to MedDiet intervention in different disease states is limited, but what has been observed is listed under each respective disease. ↓: decreased with the consumption of MedDiet. ↑: increased with consumption of MedDiet.

The present literature reveals that consuming a global MedDiet leads to alterations in the microbiome; however, the extent to which these changes occur likely depends on numerous factors. The study duration, disease risk, progression and severity, and dietary adherence potentially impact the observed microbial changes. Furthermore, smoking, alcohol consumption, physical activity, and other lifestyle behaviors may also be important determinants in influencing gut microbial composition. It is well known and widely accepted that consumers of healthy dietary patterns often engage in a variety of positive health practices, thus restricting the credibility of intervention studies as an appropriate evaluation method of the MedDiet’s microbial influence on health [92]. Further research is necessary to better identify the general outcome of MedDiet consumption on the microbiome and to identify contributing factors to microbial changes. Figure 2 provides a summary of the evidence of the previous two sections.

## 4. Conclusions, Implications, and Future Directions

In recent years, high-throughput metabolomic and microbiome analysis techniques have emerged as promising, objective tools to aid and complement the traditional epidemiology methods used to assess diet. Due to the complexity of the diet–disease relationship, assessing both the metabolome and microbiome of an individual may help provide information regarding dietary patterns in light of the information provided in Food Frequency Questionarries (FFQs), food diaries, and interviews. In addition, metabolomics can help to document compliance in dietary intervention studies, although it should be recognized that unlike in drug studies, blinding and/or randomization of the participants may be difficult in nutrition research. Currently, metabolomic literature has identified numerous metabolites related to the consumption of individual dietary components and has begun to identify metabolomic patterns in response to global dietary patterns. Identifying specific physiologically relevant small metabolites derived after the ingestion of unique diets may provide insight into health status and merits further investigation. In order for metabolites to assist in dietary assessment, they must be stable and sensitive to dietary consumption and also reflect long-term usual intake. Subtle and intermittent fluctuations in metabolite concentrations, as well as a short half-life, may not only pose analytical challenges, but also may only be indicative of recent dietary intake and not a global dietary pattern.

The current MedDiet–microbiome literature, though promising, does not provide conclusive evidence on the relative contributions of the gut microbiome on the beneficial health outcomes associated with the MedDiet [110]. Emerging evidence from the literature points to the prominent role of the microbiome in human health, although the causality and governing mechanisms remain unclear. Moreover, differences in microbial profiles appear to be dependent upon several factors including age, habitual dietary consumption, overall health, disease risk, and underlying pathology [110,111,112]. Therefore, at this time, the analysis of microbial composition, though promising, cannot be recommended as a stand-alone tool for either dietary assessment or related health outcomes. It can, however, complement and strengthen the existing tools, and it holds significant potential as a promising tool in the future. Further research that comprehensively examines the relationships between dietary patterns, microbial changes, and disease outcomes should also focus on using standardized methods and reporting processes that are transferrable across different research groups [110]. Furthermore, systemic approaches that integrate both metabolomics and the microbiome will help researchers to understand their complex interrelationships and interactions with health. The evidence shows that some metabolites (e.g., SCFA production) are mediated by the gut microbiome [40,112]. For example, the CORDIOPREV study found that changes in the metabolites in feces were accompanied by changes in the gut microbiota [101], and a recent investigation within the Malmö Offspring Study found an association between four gut microbiota genera and body mass index-predicted plasma metabolites, including glutamine and BCAAs [113]. Thus, evidence is emerging in this regard, and recent and future studies will further elucidate these relationships/associations (such as the potential effect of the microbial amino acid metabolism on obesity [114]).

It is undeniable that emerging, big data, multi-omics approaches will improve our understanding of the distinctive metabolomics/microbial characteristics that contribute to our overall health and disease risk at an individual and population level. Each method is accompanied by its own strengths and limitations, but when used together, subjective dietary assessments, metabolomic assessments, and microbiome assessments may be able to provide a much more complete picture of the diet–health relationship. This knowledge can have a significant impact on the shaping of policy and the advancement of targeted precision/personalized nutrition-based therapeutic approaches for the prevention and management of chronic diseases [110,115].

## Figures and Tables

**Table 1:** Metabolomics as an assessment of the Mediterranean diet (MedDiet) dietary pattern.

	Study Design	Participants	Dietary Pattern/Intervention	Follow-Up	Biological Sample/Metabolomics Approach/Technique	Biomarkers Identified	Main Conclusion
Playdon et al., 2017 [51]	5 nested case-control studies (within the Alpha-Tocopherol, Beta-Carotene Cancer Prevention Study)	Male Finnish smokers *n =* 1336, aged 50–69 years	HEI ^1^ 2010, aMED ^2^, HDI ^3^, and BSD ^4^	3 years	Serum Untargeted MS	HEI 2010, HDI and BSD: associated with 17, 11, and 10 identified metabolites, respectively. **aMED:** associated with 21 identifiable metabolites: 4 aminoacids (indolebutyrate, tryptophan betaine, N-methylproline, 3-Hydroxy-2-ethylpropionate); 1 carbohydrate (threitol); 2 co-factors (threonate, γ-CEHC ^5^); 3 xenobiotics (stachydrine, Phytanate, ergothionein) 11 lipids (1-myristoleoylglycerophosphocholine (14:1), Scyllo-inositol, Mead acid (20:3n -9), g-CEHC, cis-4-Decenoyl carnitine, 3-Carboxy-4-methyl-5-propyl-2-furanpropanoate, linoleate (18:2n-6), linolenate (α or γ; 18:3n-3 or 18:3n-6), chiro-inositol, 1-linoleoylglycerol, DHA, methyl palmitate	The HEI-2010, aMED, HDI, and BSD were associated with metabolites correlated with foods that are used to evaluate adherence to each score.
Vázquez-Fresno et al., 2015 [53]	Parallel-group, single-blind, multicenter, randomized, controlled feeding trial. A follow-up in the PREDIMED ^6^ study.	Clinically identified non-diabetic participants at high CVD ^7^ risk *n =* 98 aged 55–80 years	MedDiet ^8^ + EVOO ^9^ (*n =* 41) (MedDiet + nuts (*n =* 27) LFD ^10^ (*n =* 30)	3 years	Urine (baseline, 1 year, and 3 year of the intervention) Untargeted NMR	**MedDiet**: carbohydrates (3-HB **^11^**, citrate, and cisaconitate), creatine, creatinine, amino acids (proline, N-acetylglutamine, glycine, branched-chain amino acids, and derived metabolites), lipids (oleic and suberic acids), and microbial cometabolites (PAGN **^12^** and p-cresol) **LFD**: hippurate, TMAO ^13^, anserine, histidine and derivates (3-MH **^14^**, 1-MH, carnosine, anserine), and xanthosine.	The MedDiet groups had distinct metabolic profiles compared to the baseline and control group related to carbohydrate and lipid metabolism, amino acids, and microbial cometabolites (PAGN and p-cresol)
Bondia-Pons et al., 2015 [52]	Randomized controlled dietary intervention	Individuals with high BMI and at least two features of metabolic syndrome. *N =* 72	RESMENA **^1^^5^** diet (*n =* 47) (based on MedDiet). 7 meals/day. (40% CHO **^16^**, 30%protein, 30% lipid). Control diet (*n* = 45) (American Heart Association guidelines). 5 meals/day (55% CHO 55%, 15% protein, 30% lipid)	6-month (2-month nutritional learning followed by a 4-month self-control period)	Plasma Non-targeted MS-Liquid	Lipids, mainly phospholipids and lysophospholipids. lactic acid, L-isoleucine, alloisoleucine, hydroxyvaleric acid, hypaphorine, paraxanthine, hippuric acid, furancarboxylic acid, LysoPC **^17^** (14:0), LysoPC (20:5), LysoPC (16:1), LysoPC (22:6), LysoPE (20:4), LysoPE **^18^** (18:2), LysoPC (16:0), Linoleamide, LysoPC (20:3), LysoPE (18:1), LysoPC (18:1), LysoPC (20:4), Eicosapentaenoic acid, LysoPC (15:0), Lithocholic acid, Oleamide, 1-Monopalmitin, LysoPC (18:0), GPL **^19^** containing (18:2), Palmitic acid, PC **^20^**, and PE **^21^**	The major discriminative markers between the two groups were the plasmalogen PC (P **^22^**-18:1/20:3) after 2 months and palmitic acid after 6 months.
González-Guardia et al., 2015 [55]	Randomized, crossover	Men (*n =* 5) and women (*n =* 5) aged 65 years or older. *n =* 10	MedDiet+ 200mg/d CoQ ^23^; MedDiet without CoQ Western diet rich in SFA **^24^**; Low-fat, high-carbohydrate diet enriched in n-3 PUFA^25^.	4 weeks	Urine and plasma Targeted NMR	CoQ and β-carotene plasma levels and isoprostanes urinary levels were determined. Higher levels of hippurate and lower levels of phenylacetylglycine were found when comparing the MedDiet + CoQ and the SFA.	The MedDiet supplemented with CoQ is associated with increased levels of excreted hippurate and decreased levels of phenylacetylglycine compared with a SFA-rich diet.
Kakkoura et al., 2017. [56]	Cross-sectional study.	Greek-Cypriot control women who have previously participated in the population-based case- control study of BC, MASTOS [61]. *n =* 564	MedDiet (highest and lowest adherence to MedDiet)	N/A	Serum Targeted. UPLC-MS/MS	5-MTHF **^26^**, riboflavin, FMN **^27^**, PA, methionine, methionine sulfoxide, SAM **^28^**, SAH **^29^**, total HCY **^30^**, cystathionine, total cysteine, γ-glu-cys, total GSH **^31^**, and α-hydroxybutyrate.	Higher adherence to the MedDiet was associated with an increase in antioxidant-related metabolites, 5-MTHF.

^1^ HEI: Healthy Eating Index. ^2^ aMED: Mediterranean diet score. ^3^ HDI: the WHO Healthy Diet Indicator. ^4^ BSD: the Baltic Sea Diet. ^5^ CEHC: carboxyethyl hydroxychroman. ^6^ PREDIMED: Prevención con Dieta Mediterránea. ^7^ CVD: cardiovascular disease. ^8^ MedDiet: Mediterranean diet. ^9^ EVOO: extra virgin olive oil. ^10^ LFD: a control, low-fat diet. ^11^ HB: hydroxybutyrate. ^12^ PAGN: phenylacetylglutamine. ^13^ TMAO: Trimethylamine N-oxide. ^14^ MH: Methylhistidine^. 15^ RESMENA: Metabolic Syndrome Reduction in Navarra. ^16^ CHO: Carbohydrates. ^17^ LysoPC: lysophosphatidylcholine. ^18^ LysoPE: lysophosphatidylethanolamine. ^19^ GPL: glycerophospholipid. ^20^ PC: phosphatidylcholine. ^21^ PE: phosphatidylethanolamine. ^22^ P: plasmalogen. ^23^ CoQ: coenzyme Q. ^24^ SFA: Saturated fatty acids. ^25^ PUFA: polyunsaturated fatty acids. ^26^ 5-MTHF: L-Methylfolate. ^27^ FMN: flavin mononucleotide. ^28^ SAM: S-adenosylmethionine. ^29^ SAH: S-adenosylhomocysteine. ^30^ HCY: Homocysteine. ^31^ GSH: glutathione. MS: Mass spectrometry; DHA: Docosahexaenoic Acid; NMR: nuclear magnetic resonance; BMI: Body mass index. 5-MTHF: 5-Methylfolate.

**Table 2:** Metabolomics, the Mediterranean diet pattern, and its association with CVD and T2D.

Author	Study Design	Study Population	Dietary Pattern/Intervention	Follow-Up	Biological Sample/Metabolomics Approach	Metabolites Examined	Main Conclusion
Guasch-Ferré et al., 2016 [66]	Case–cohort study within the PREDIMED ^1^ study	Participants aged 55–80 years at high risk of CVD ^2^. *n =* 980 (229 cases CVD cases).	MedDiet ^3^ groups: (MedDiet + EEVO ^4^ and MedDiet + mixed nuts. Control group: low-fat diet.	4.8 years	Plasma. (baseline and after 1 year) Targeted	28 acylcarnitines: short-chain acylcarnitines (C2–C7), medium-chain acylcarnitines (C8–C14), and long-chain acylcarnitines (C16–C26).	An increased level of acylcarnitines metabolic profiles is independently associated with total CVD risk and risk of stroke. MedDiet interventions may attenuate the association between acylcarnitines and CVD risk.
Guasch-Ferré et al., 2017 [67]	Case–cohort study within the PREDIMED study	Participants aged 55–80 years at high risk of CVD. *n =* 980 (229 CVD cases)	MedDiet groups: (MedDiet + EEVO and MedDiet + mixed nuts. Control group: low-fat diet.	4.8 years	Plasma (baseline and after 1 year) Targeted	Metabolites of the choline pathway: TMAO ^5^, betaine, choline, phosphocholine, and a-glycerophosphocholine. A choline metabolite score was created.	The baseline choline metabolite score was associated with increased risk of CVD. The one-year changes in plasma metabolites were not significantly associated with CVD. The participants in the highest metabolite score quartile and assigned to low-fat diets had higher risk of CVD than those in the lowest metabolite quartile and in the MedDiet group. No significant interaction was found between the continuous choline score, the betaine/choline ratio, and the intervention group and CVD
Yu et al., 2017 [68]	Case–cohort study within the PREDIMED study, controlled trial	Participants aged 55–80 at high risk of CVD. *n =* 985 (231 CVD cases)	MedDiet groups: (MedDiet + EEVO and MedDiet + mixed nuts. Control group: low-fat diet.	4.7 year	Plasma (baseline and after 1 year) Targeted	Tryptophan, kynurenine, kynurenic acid, 3-hydroxyanthranilic acid, and quinolinic acid concentrations. A KRS ^6^ was created.	The positive association between the KRS and CVD risk is stronger in the control group, indicating that the MedDiet may attenuate the effect of a high KRS score.
Toledo et al., 2017 [77]	Case–cohort study within the PREDIMED study	Participants aged 55–80 years at high risk of CVD. *n =* 983 (230 CVD cases)	MedDiet groups: (MedDiet + EEVO and MedDiet + mixed nuts. Control group: low-fat diet.	4.8 years	Plasma (baseline and after 1 year) Untargeted lipidome	202 lipid species	The baseline concentrations of cholesterol esters (CEs) were inversely associated with CVD. The MedDiet interventions resulted in changes in the lipidome at 1 year; however, they were not found to be associated with subsequent CVD risk. Lipid metabolites with a longer acyl chain and a higher number of double bonds at the baseline were significantly and inversely associated with the risk of CVD.
Wang et al., 2017 [69]	Case–cohort study within the PREDIMED study	Participants aged 55–80 years at high risk of CVD. *n =* 980 (230 CVD cases)	MedDiet groups: (MedDiet + EEVO and MedDiet + mixed nuts. Control group: low-fat diet.	≤7.4 years	Plasma (baseline and after 1 year) Targeted	4 different ceramides: ceramide (d ^7^ 18:1/16:0), ceramide (d18:1/22:0), ceramide (d18:1/24:0), and ceramide (d18:1/24:1). A ceramide score was calculated.	The ceramide score was positively associated with the risk of CVD. The MedDiet may alleviate the potential negative effects of increased plasma ceramide levels on CVD.
Zheng et al., 2017 [70]	Case–cohort study within the PREDIMED study	Participants aged 55–80 years at high risk of CVD. *n =* 980 (788 subcohort, 192 incident external cases)	MedDiet groups (intervention diets): (MedDiet + EEVO and MedDiet + mixed nuts. Control group: low-fat diet.	4.8 years	Plasma (baseline and after 1 year) Targeted	Glu14 ^8^ Gln15 ^9^, Glu/Gln ratio No significant effect of the intervention on one-year changes in the metabolites. No effect of the changes themselves on the CVD risk was apparent.	A positive association between Glu levels and CVD risk (43% increased risk) and a negative association between Gln/Glu and risk of CVD (25% decreased risk) were found. The interventions effectively lowered CVD risk for the participants with high baseline Glu, while no effects were found among the participants with low baseline Glu.
Razquin et al., 2018 [71]	Unstratified case–cohort design within the PREDIMED study.	*n =* 983 participants (233 CVD cases).	MedDiet groups: (MedDiet + EEVO and MedDiet + mixed nuts. Control group: low-fat diet.	4.8 years	Plasma (baseline and after 1 year)	Lipid group A: PC ^10^ (PCs, LysoPC^11^s and PC-plasmalogens with ≥5 double bonds); CE ^12^ with N3 double bonds; and TAG ^13^ with ≥52 carbon atoms containing ≥6 double bonds. Lipid group B: MAG ^14^; DAG ^15^; short-chain, TAGs containing ≤4 double bonds; PEs ^16^ except those with saturated fatty acids; hydroxyPC. PC, CE, long-chain TAG, MAG and DAG, short-chain TAG, PE, and Hpc ^17^ scores were calculated.	The metabolites from lipid group A were inversely associated with CVD; the metabolites from lipid group B were directly associated with CVD. The baseline phosphatidylethanolamines (PEs) and their one-year changes tended to be associated with higher CVD risk. No significant effect of the MedDiet intervention was found on the metabolite scores.
Ruiz-Canela et al., 2016 [78]	Case–cohort study within the PREDIMED study	*n =* 970 (226 CVD cases)	MedDiet groups: (MedDiet + EEVO and MedDiet + mixed nuts. Control group: low-fat diet.	4.8 years	Plasma (baseline and after 1 year)	BCAAs	Higher concentrations of baseline BCAAs were associated with increased risk of CVD. No significant effect of the intervention on one-year changes in BCAAs or any association between one-year changes in BCAAs and CVD were observed.
Yu et al., 2017 [73]	Case–cohort study within the PREDIMED study	*n =* 984 (231 CVD cases)	MedDiet groups: (MedDiet +EEVO and MedDiet + mixed nuts. Control group: low-fat diet.	4.7 years	Plasma (baseline and after 1 year)	arginine, ornithine, citrulline, ADMA ^18^, symmetric dimethylarginine (SDMA ^19^), and NG-monomethylarginine (NMMA ^20^)	A higher baseline arginine/asymmetric dimethylarginine ratio was associated with lower CVD incidence. No significant modification by the MedDiet after one-year intervention was observed.
Guasch-Ferre et al., 2018 [79]	Case–cohort study in the PREDIMED study	*n =* 892 participants (251 T2D cases)	MedDiet groups: (MedDiet + EEVO and MedDiet + mixed nuts. Control group: low-fat diet	3.8 years	Plasma (baseline and after 1 year)	Short-chain acylcarnitines (C2–C7), medium-chain acylcarnitines (C8–C14), and long-chain acylcarnitines (C16–C26).	The acylcarnitines profile, specifically short- and long- chain acylcarnitines, was significantly associated with a higher risk of T2D.
Yu et al., 2018 [73]	Case–cohort study in the PREDIMED study	*n =* 892 participants (251 T2D cases)	MedDiet groups: (MedDiet + EEVO and MedDiet + mixed nuts. Control group: low-fat diet	1 year	Plasma	Arginine, citrulline, ornithine, ADMA, SDMA, and NMMA	The one-year changes in arginine and the arginine/ADMA ratio were negatively associated with the risk of T2D ^21^. Positive changes in ornithine and citrulline and negative changes in SDMA and GABR were inversely associated with concurrent changes in HOMA-IR ^22^ The MedDiet significantly modified the association between one-year changes in NMMA and T2D risk.
Ruiz-Canela et al., 2018 [72]	Case–cohort study in the PREDIMED study	*n =* 945 participants (251 T2D cases)	MedDiet groups: (MedDiet + EEVO and MedDiet + mixed nuts. Control group: low-fat diet	3.8 years	Plasma (baseline and after 1 year)	The baseline BCAA ^23^ (leucine, isoleucine and valine) and AAA ^24^ (phenylalanine and tyrosine) scores were associated with a higher risk of T2D. Increases in the BCAA score after one year were associated with higher T2D risk only in the control group.	The MedDiet rich in EVOO significantly reduced the levels of BCAA and attenuated the positive association between plasma BCAA levels and T2D incidence.
Papandreou et al. 2018 [76]	Case–cohort study in the PREDIMED study	*n* = 945 participants (251 T2D cases)	MedDiet groups: (MedDiet + EEVO and MedDiet + mixed nuts. Control group: low-fat diet	3.8 years	Plasma (baseline and after 1 year)	TMAO, L-carnitine, betaine, LPC and LPE species, phosphocholine, α-glycerophosphocholine, and choline. Higher baseline concentrations of TMAO, L-carnitine, betaine, α-glycerophosphocholine, and several LPC ^25^ and LPE ^26^ species were associated with a lower risk of T2D development.	There was no significant difference in the association of most of the one-year changes in the metabolites with T2D risk in the MedDiet intervention and control groups. The intervention diets did not appear to significantly change the study metabolite levels during the intervention.
Razquin et al. (2018) [74]	Case–cohort study in the PREDIMED study	*n =* 942 participants (250 T2D cases)	MedDiet groups: (MedDiet + EEVO and MedDiet + mixed nuts. Control group: low-fat diet	3.8 years	Plasma (baseline and after 1 year)	The baseline TAGs, DAGs, and PEs were positively associated with T2D risk. TAGs with odd-chain fatty acids showed inverse associations with T2D after adjusting for total TAGs.	The one-year changes in the baseline metabolites associated with T2D were not significant. The changes in LP ^27^, PC-PL ^28^, SM ^29^, and CE scores showed no apparent mediating effects.
Yu et al., 2018 [75]	Case–cohort study in the PREDIMED study	*n =* 892 participants (251 T2D cases)	MedDiet groups: (MedDiet + EEVO and MedDiet + mixed nuts. Control group: low-fat diet	3.8 years	Plasma (baseline and after 1 year) Targeted	Tryptophan, kynurenine, kynurenic acid, 3-hydroxyanthranilic acid, and quinolinic acid concentrations. A KRS score was created.	The baseline tryptophan and one-year increases in quinolinic acid were positively associated with incident T2D. No effect of the MedDiet was observed.

^1^ PREDIMED: Prevención con Dieta Mediterránea. ^2^ CVD: cardiovascular disease. ^3^ MedDiet: Mediterranean diet. ^4^ EVOO: extra virgin olive oil. ^5^ TMAO: trimethylamine N-oxide. ^6^ KRS: kynurenine risk score. ^7^ d: shorthand notation of sphingolipids refer to 1,3 dihydroxy. ^8^ Glu: glutamate. ^9^ Gln: glutamine. ^10^ PC: phosphatidylcholines. ^11^ LysoPC: lysophosphatidylcholine. ^12^ CE: cholesterol esters. ^13^ TAG: long-chain triacylglycerols. ^14^ MAG: monoacylglycerols. ^15^ DAG: diacylglycerols. ^16^ PE: phosphatidylethanolamine. ^17^ hPC: hydroxyPC ^18^ ADMA: asymmetric dimethylarginine. ^19^ SDMA: symmetric dimethylarginine. ^20^ NMMA: N-monomethyl-l-arginine. ^21^ T2D: type 2 diabetes. ^22^ HOMA-IR: homeostatic model assessment of insulin resistance. ^23^ BCAA: branched-chain amino acids. ^24^ AAA: aromatic amino acids. ^25^ LPC: lyso- phosphatidylcholine. ^26^ LPE: lyso-phosphatidylethanolamine. ^27^ LP: lysophospholipids. ^28^ PC-PL: phosphatidylcholine-plasmalogens. ^29^ SM: sphingomyelins.

**Table 3:** Effects of a Mediterranean diet on microbiome composition.

Author	Study Design	Study Population	Dietary Pattern/Intervention	Follow-up	Sample	Microbiota Observed	Results/Conclusion
Gutierrez-Diaz et al., 2016 [92]	Cross-sectional	Adults with a non-declared pathology; *n =* 31 (23 females, 8 males, mean age of 42.1 years	MedDiet ^1^ score (0–8 points; > 4 = High adherence)	N/A	Stool	Bifidobacterium, Faecalibacterium, Tenericutes, Dorea, Rikenellaceae, Alistipes, Ruminococcus (Lechnospiraceae family), Coprococcu, Bacteroidetes, Prevotel-lacceae, Prevotella, and Firmicutes	The MedDiet score was associated with a higher abundance of Bacteroidetes, Prevotel-laceae, and Prevotella and a lower concentration of Firmicutes and Lachnospiraceae.
Gutierrez-Diaz et al., 2017 [102]	Cross-sectional	Healthy men (*n* = 20) and women (*n* = 54) older than 50 years of age	MedDiet	N/A	Stool	Akkermansia, Bacteroides-Prevotella-Porphiromonas, Bifidobacterium, Clostridium cluster XIVa, Lactobacillus group, and F. prausnitzii	Higher levels of Clostridium cluster XIVA and *F*. *prausnitzii* were found in subjects with MDS ^2^ scores ≥4 and were positively correlated with fecal concentrations of benzoic and 3-hydroxyphenylacetic acids and the intake of polyphenols and fibers.
De Filippis et al., 2016 [101]	Cross-sectional study	Healthy Volunteers *n* = 153	MedDiet Vegan Vegetarian Omnivore	N/A	Stool, urine	Lachnospira, Prevotella, Roseburia, and Ruminococcus	Plant-based diets appear to increase fecal SCFAs, while Prevotella specifically was associated with fiber-degrading Firmicutes. Higher urinary trimethylamine oxide levels were found to be higher in those with lower MedDiet adherence. Beneficial microbiome-related metabolic profiles were associated with the increased consumption of plant-based foods, consistent with a MedDiet.
Garcia-Mantrana et al., 2018 [103]	Cross-sectional study	Healthy individuals *n =* 27, mean age 39.5 years	MedDiet	N/A	Stool samples	Enterobacteriaceae family, *Bifidobacterium* group, *Bacteroides-Prevotella-Porphyromonas* group, *Bacteroides fragilis* group, *Blautia coccoides* group, *Methanobrevibacter smithii*, and *Faecalibacterium prausnitzii*	A higher ratio of Firmicutes–Bacteroidetes was related to lower adherence to the MedDiet, and greater presence of Bacteroidetes was associated with lower animal protein intake. Better adherence to the MedDiet was associated with significantly higher levels of total SCFA ^3^.
Shankar et al., 2017 [96]	Comparative cross-sectional stud	Healthy Egyptian male teenagers *n =* 28, mean age 13.9 years Healthy American male teenagers *n =* 14, mean age 12.9 years	MedDiet Western diet	N/A	Stool	Egyptian: Gammaproteobacteria, Methanobacteria, Prevotella, Megasphaera, Eubacterium, Mitsuokella, Catenibacterium U.S.: Clostridia, Verrucomicrobia, Bacteroides, Ruminococcus, Coprococcus, Blautia, Bilophila, Akkermansia, and Faecalibacterium,	Egyptian gut microbial communities belonged to Prevotella in all the subjects with increased polysaccharide-degrading microbes and end products of polysaccharide fermentation. United States (US) gut microbial communities mostly belonged to Bacteroides with increased proteolytic microbes and end products of protein and fat metabolism.
Djuric et al., 2018 [100]	Randomized control trial	*n* = 88 baseline samples *n* = 82 post-intervention (men and women) Mean age 53 years	MedDiet (30% kcals form fat, PUFA/SAT/MUFA ^4^ ratios of 1:2:5, foods high in n-3 fatty acids 2x/week, 3 servings/day whole grains, 7–9.5 cup s/day F ^5^+V ^6^) including at least one cup dark green or orange F or V). Healthy Eating diet (5.5 cup servings/day F + V, 3 s/day whole grains, <10% kcals from sat. fat.	6 months	Blood, colon biopsy	Firmicutes, Proteobacteria, Lachnospiraceae, Blautia, Roseburia, Prevotella, and Bacteroides,	A total of 11 operational taxonomic units were significantly associated with increased serum carotenoid levels. The Bacteria in the colonic mucosa was resistant to change after both diet interventions The intestinal microbiota did not show significant changes after 6 months of diet intervention; however, an abundance of specific OTUs ^7^ was significantly associated with serum carotenoid concentrations at the baseline, suggesting that long-term dietary exposures may have more of an influence on bacteria in the colonic mucosa.

^1^ MedDiet: Mediterranean diet.^2^ MDS: Mediterranean Diet Score. ^3^ SCFA: Short Chain Fatty Acids. ^4^ PUFA/SAT/MUFA: polyunsaturated fatty acids/saturated fatty acids/mono-unsataurated fatty acids: ^5^ F: Fruits. ^6^ V: Vegetables, ^7^ OTUs: Operational Taxonomic Unit.

**Table 4:** Mediterranean diet effects on the microbiomes of diseased subjects.

	Design	Participant Characteristics	Dietary Pattern/Treatment	Length	Sample	Microbiota Observed	Results/Conclusion
Haro et al., 2016 [109]	Randomized control trial	*n =* 138 with metabolic syndrome (MetS) and *n =* 101 without MetS; male and female patients within the CORDIOPREV study with CHD ^1^, who had their last coronary event over 6 months before enrolling, in addition to conventional treatment for CHD,	MedDiet group:35% fat (22% MUFA, 6% PUFA, 7% SAT). Low-fat high-complex carb (LFHCC) diet group: 28% fat (12% MUFA, 8% PUFA, 8% SAT).	2 years	Stool, blood	Bacteroides, Eubacterium, Lactobacillus, Bacteroides fragilis group, Parabacteroides distasonis, Bacteroides thetaiotaomicron, Faecalibacterium prausnitzii, Fusobacter- ium nucleatum, Bifidobacterium longum, Bifidobacterium adolescentis, Ruminococcus flavefaciens subgroup, and Eubacterium rectale	The long-term consumption of the Mediterranean diet partially restores the population of *P. distasonis*, *B. thetaiotaomicron*, *F. prausnitzii*, *B. adolescentis and B. longum* in MetS patients although MetS persists.
Haro et al., 2016 [104]	Randomized control trial	*n*= 20, 40 total samples collected (20 at the baseline, 20 post-intervention) from obese men with CHD within the CORDIOPREV study	MedDiet group: 35% fat (22% MUFA, 6% PUFA, 7% SAT). Low-fat high-complex carb (LFHCC) diet group: 28% fat (12% MUFA, 8% PUFA, 8% SAT).	1 year	Blood, stool	Bacteroides, Prevotella, unknown Lachnospiraceae, Faecalibacterium, unknown Clostridiales, unknown Ruminococcaceae, Oscillospira, Parabacteroides, and unknown Bacteroidales	Both diet changes increased insulin sensitivity and appeared to exert protective effects on the development of T2DM ^2^ based off of specific changes in gut microbiota. Changes in feces include mostly amino acids, peptides, and shingolipid metabolism, which may be linked to changes occurring in the gut microbiota.
Haro et al., 2017 [107]	Randomized control trial	*n* = 33 obese patients with severe MetS–OB ^3^, *n* = 32 obese patients without non-MetS–OB, and 41 non-obese subjects (non-MetS–non-OB).	MedDiet group: 35% fat (22% MUFA, 6% PUFA, <10% SAT). Low-fat diet group: <30% total fat (<10% SAT., 12–14% MUFA, 6–8% PUFA).	2 years	Stool	Actinobacteria, Bacteroidetes, Firmicutes, Bacteroides, Prevotella, Roseburia, Faecalibacterium, Ruminococcus, Streptococcus, Clostridium, P. distasonis, and F. prausnitzii	Both diets were associated with partially restored gut microbiome dysbiosis, converting MetS-OB microbiota patterns to microbiota patterns similar to those found in (metabolically) healthy people, after 2 years of nutrition intervention in participants with coronary heart disease. The degree of participants’ metabolic dysfunction may alter the effectiveness of nutrition therapy.
Marlow et al., 2014 [83]	Non-randomized trial	*n =* 8 Crohn’s patients with no history of bowel surgery who were not taking prednisone or similar anti-inflammatory medication and had no changes in medication over the last 3 months.	MedDiet	6 weeks	Stool, blood	Firmicutes, Bacteroidetes, Actinobacteria, Proteo- bacteria, Fusobacteria, and Verrucomicrobia	The Mediterranean-inspired diet appeared to benefit the health of people with Crohn’s disease. The participants showed a trend for reduced markers of inflammation and normalization of the microbiota; however, the changes were not significant.
Mitsu et al., 2017 [108]	Cross-sectional	*n =* 120 Men and women, age 18–65 years	MedDiet Tertiles of adherence based on MedDiet score: Low tertile score = 19–30 (*n* = 31) Medium tertile score = 31–33 (*n* = 29) High tertile score = 34–41 (*n* = 40)	N/A	Stool	*E. coli*, bifidobacteria, and *Candida albicans*	The findings support a link between MedDiet adherence and the gut microbiota profile. Those with high adherence had lower *E. coli* counts, a higher bifidobacterial/E. coli ratio, and increased levels and prevalence of *Candida albicans* when compared to those with low adherence
Garcia-Mantrana et al., 2018 [103]	Cross-sectional study	Healthy individuals *n =* 27, mean age 39.5 years	MedDiet		Stool samples	Enterobacteriaceae family, *Bifidobacterium* group, *Bacteroides-Prevotella-Porphyromonas* group, *Bacteroides fragilis* group, *Blautia coccoides* group, *Methanobrevibacter smithii*, and *Faecalibacterium prausnitzii*	*Butyricimonas*, *Desulfovibrio*, and *Oscillospira* genera were associated with a BMI of <25 and the genus *Catenibacterium* was associated with a higher adherence to the MedDiet.

^1^ CHD: coronary heart disease.^2^ T2DM: type II diabetes.^3^ OB: Obese subjects.

## Supplementary Materials

The following are available online at http://www.mdpi.com/2072-6643/11/1/207/s1, Table S1: Main findings and evidence from metabolites associated with food.
