# Supplementary material for: Metabolomics and Microbiomes as Potential Tools to Evaluate the Effects of the Mediterranean Diet
Source: Nutrients. 2019 Jan 21;11(1):207. doi: 10.3390/nu11010207 (PMC6356665; doi:10.3390/nu11010207)
Supplement: Supplementary file 1 [file nutrients-11-00207-s001.pdf]

Table S1. Main findings and evidence from metabolites associated with food (higher levels of metabolites are associated with higher levels of food item consumption/dietary pattern adherence unless otherwise specified).

Metabolite	Food Item/Dietary Pattern
TMAO	Meat products [1]; omnivorous diet [2]; seafood [3]; Nordic diet [3]; low glycemic load diet [4]; low dairy intake [5]; a pattern that is high in meat, refined bread, and butter and low in vegetables, whole-meal bread, and fruits (cluster 3) [6]; low-fat diet [7,8]; and a diet that is most concordant with the WHO healthy eating guidelines [9]
Dimethylamine	Omnivorous diet [2]; a diet that is most concordant with the WHO healthy eating guidelines [9]
2,6-dihydroxybenzoic acid	High-fiber diet [10]
2-aminophenol sulfate	High-fiber diet [10]
Hippuric acid	Dietary fiber intake [11]; tea consumption [3,12,13]; phytochemical diet [14]; Nordic diet [3]; a diet high in whole grains, fatty fish, and blueberries [15]; whole grain [15]
Hippurate	Low dairy consumption [5]; low-fat diet [8]; a diet that is most concordant with the WHO healthy eating guidelines [9]; low glycemic index diet [7]; a healthy cluster: higher intakes of breakfast cereals, low fat and skimmed milks, potatoes, fruit, fish, and fish dishes [16].
Kynurenate	Low glycemic load diet [4]
Valine	Protein intake [17]; high in potatoes, dairy products, and cornflakes intake dietary pattern [18]; fish eaters [19]; vegetarians [19]; Mediterranean diet [8].
Phenylalanine	Protein intake [17]; high-fat meal with whey protein isolate [20]; coffee consumption negatively associated with phenylalanine in men [21]; Western dietary pattern [22]; omnivorous diet [2];
Tyrosine	Protein intake [17]; fish eaters [19]; vegetarians [19]
Glutamine	Inversely associated with protein intake [17]
Tryptophan	High-fat meal with whey protein isolate [20]; fish eaters [19]; vegetarians [19]; a healthy cluster: higher intakes of breakfast cereals, low fat and skimmed milks, potatoes, fruit, fish, and fish dishes [16].
Kynurenine	High-fat meal with whey protein isolate [20]
Theobromine metabolites	Cocoa consumption [23]
Polyphenol metabolites	Cocoa consumption [23]; consumption of poly-phenol rich foods (coffee, tea, red wine, citrus fruit, apples, pears, and chocolate) [24]
Tartrate	Wine polyphenol consumption [25]
Hydroxyphenylvaleric	Flavan-3-ols from almond skin [26]
Hydroxyphenylpropionic	Flavan-3-ols from almond skin [26]
Hydroxyphenylacetic acids	Flavan-3-ols from almond skin [26]
Betonicine	High polyphenol orange juice [27]
Stachydrine	High polyphenol orange juice [27]
Methyl glucopyranoside	High polyphenol orange juice [27]
Dihydroferulic acid	High polyphenol orange juice [27]
Galactonate	High polyphenol orange juice [27]
Proline	Citrus intake [28,29], Mediterranean diet [8].
Betaine	Citrus intake [20,29]; a healthy cluster: higher intakes of breakfast cereals, low fat and skimmed milks, potatoes, fruit, fish, and fish dishes [16]
S-methyl-L-cysteine sulfoxide	Cruciferous vegetables intake [30]

Creatine	High-lycopene tomato sauce [31]; Mediterranean diet [8]; a diet that is least concordant with the WHO healthy eating guideline and has high intake of red meat [9]
Creatinine	High-lycopene tomato sauce [31]; high dairy consumption [5], Mediterranean diet [8]; an unhealthy cluster: higher intakes of chips/processed potatoes, meat products, savory snacks, and high-energy beverages [7]
Leucine	High-lycopene tomato sauce [31]; refined wheat bread [32]; Western dietary pattern [22]; high potatoes, dairy products, and cornflakes intake dietary pattern [18]; fish eaters [19]; vegetarians [19]; Mediterranean diet [8].
Choline	High-lycopene tomato sauce [31]
Methionine	High-lycopene tomato sauce [31]; high potatoes, dairy products, and cornflakes intake dietary pattern [18]; fish eaters [19]; vegetarians [19]
Acetate	High-lycopene tomato sauce [31]
Ascorbic acid	Normal-lycopene content tomato sauce [31]
Lactate	Normal-lycopene content tomato sauce [31]
Pyruvate	Normal-lycopene content tomato sauce [31]
Isoleucine	Normal-lycopene content tomato sauce [31]; refined wheat bread [32]; high potatoes, dairy products, and cornflakes intake dietary pattern [18]; Mediterranean diet [8].
Alanine	Normal-lycopene content tomato sauce [31]; low-fat diet [7]
Sphingomyelins	Coffee [33]
Acylcarnitines	Coffee (negative association with long- and medium chain acylcarnitines) [33], Western dietary pattern (positive association with short-chain acylcarnitines) [22], high butter intake and low margarine intake dietary pattern [18], hypocaloric diet (acylcarnitine C9) [17], meat intake (C0, C4, C5) [19], prudent pattern (medium- to long- chain) [22]
Diacylphosphatidylcholine	Coffee consumption (C32:1, negatively association) [21], dietary component: high poultry, fish, rice, vegetables, fruit, chocolate, flaked oat, cheese, milk, curds, and low meat and sausages [34]
Acylalkyl-phosphatidylcholines	Coffee consumption (positive associated with C34:3, C40:6, and C42:5 in women) [21], high butter intake and low margarine intake dietary pattern [18], dietary component: high poultry, fish, rice, vegetables, fruit, chocolate, flaked oat, cheese, milk, curds, and low meat and sausages [34]
Phosphatidylcholines	High red meat and fish, and low whole-grain bread and tea dietary pattern [18], hypocaloric diet (phosphatidylcholine-dyacil C38:6) [17], Mediterranean diet (P-18:1, 20:3) [35]
Methylxanthines	Coffee [36–38]
Methylated forms of hydroxycinnamates	Coffee [36–38]
Hydroxytyrosol	Olive oil consumption [39], phenolic content of food [40], alcohol consumption [39]
3-(3,5-dihydroxyphenyl)-1-propanoic acid sulfate	Whole-grain rye bread intake [41]
Enterolactone glucuronide	Whole-grain rye bread intake [41]
Azelaic acid	Whole-grain rye bread intake [41]
2-aminophenol sulfate	Whole-grain rye bread intake [41]
Lysophosphatidylcholine	Full-fat dairy [42], high butter intake and low margarine intake dietary pattern [18]
Lyso-platelet activating factor	Full-fat dairy [42]
Phospholipid fatty acids	Full-fat dairy [42]

Citrate	High dairy consumption [5], lactovegetarian diet [2], Mediterranean diet [8], a healthy cluster: higher intakes of breakfast cereals, low fat and skimmed milks, potatoes, fruit, fish and fish dishes [16]
Hexose	High red meat and fish, and low whole-grain bread and tea dietary pattern [18].
Hydroxy-sphingomyelin	High butter intake and low margarine intake dietary pattern [18]
Glycerophospholipids	Meat eaters [19]
Sphingolipids	Meat eaters [19]
Lysine	Fish eaters [19]; vegetarians [19]
p-Hydroxyphenylacetate	Vegetarian diet [19]
Methylhistidine	Omnivorous diet [2]; low-fat diet [8], a diet that is most concordant with the WHO healthy eating guidelines (1-methylhistidine and 3-methylhistidine) [9]
Phenylacetylglutamine	Vegetables [6]; high vegetables, fish, and whole-grain breads (cluster 1) [6];
O-acetylcarnitine	Red meat [6]; a pattern that is high in meat, refined bread, butter and low in vegetables, whole-meal bread, and fruits (cluster 3) [6], a diet that is least concordant with the WHO healthy eating guidelines and has high intake of red meat [9]
Glycine	High vegetables, fish, and whole-grain breads (cluster 1) [6], Mediterranean diet [8].
Acetoacetate	High vegetables, fish, and whole-grain breads (cluster 1) [6]
N,Ndimethylglycine	A pattern that is high in meat, refined bread, butter and low in vegetables, whole-meal bread, and fruits (cluster 3) [6].
Proline betaine	Citrus intake [14]
Sulforaphane	Phytochemical diet [14]
Genistein,	Phytochemical diet [14]
Daidzein	Phytochemical diet [14]
Equol	Phytochemical diet [14]
Glycitein	Phytochemical diet [14]
O-desmethylangolensin	Phytochemical diet [14]
Enterolactone	Phytochemical diet [14]
Trigonelline	Phytochemical diet [14]
Hydroquinone-glucuronide	Nordic diet [3]
3,4,5,6-tetrahydrohippurate	Nordic diet [3]
Glucuronidated alk(en)ylresorcinols	A diet high in whole grains, fatty fish, and bilberries [15]; whole-grain [15]
Furan fatty acids	A diet high in whole grains, fatty fish and bilberries [15]; fish intake [15]
Hydroxybutyrate	Mediterranean diet (3-hydroxybutyrate) [8]; very-low-carbohydrate diet (β-hydroxybutyrate) [7]; a healthy cluster: higher intakes of breakfast cereals, low fat and skimmed milks, potatoes, fruit, fish, and fish dishes (3-hydroxybutyrate) [16]
Cisaconitate	Mediterranean diet [8]
N-acetylglutamine	Mediterranean diet [8]
Oleic acids	Mediterranean diet [8]
Suberic acids	Mediterranean diet [8]
Histidine	Low-fat diet [8]
Carnosine	Low-fat diet [8]
Anserine	Low-fat diet [8]; a healthy cluster: higher intakes of breakfast cereals, low fat and skimmed milks, potatoes, fruit, fish, and fish dishes [16]
Xanthosine	Low-fat diet [8]
Nacetyl-S-methyl-cysteine-sulfoxide	Diet 1, which is most concordant with the WHO healthy eating guidelines [9]
Carnitine	A diet that is least concordant with the WHO healthy eating guidelines and has high intake of red meat [9]; very-low-carbohydrate diet [7]
Triacylglycerol	Low-fat diet (C54:5) [7]

Asparagine	Very-low-carbohydrate diet [7]
Cholesteryl esters	Very-low-carbohydrate diet [7]
Propionate	Very-low-carbohydrate diet [7]
Sorbitol	Very-low-carbohydrate diet [7]
4-pyridoxate	Low-fat diet [7]
Triacylglycerides (TG)	Low-fat diet (certain TGs) [7]; low glycemic index diet (certain TGs) [7]
Allantoin	Low-fat diet [7]
2-aminoadipate	Low-fat diet [7]
Serine	Low-fat diet [7]
Cytosine	Low glycemic index diet [7]
Hydroxyproline	Low glycemic index diet [7]
5-aminolevulinic acid	Low glycemic index diet [7]
Pipecolic acid	Low glycemic index diet [7]
N-phenylacetylglutamine	A healthy cluster: higher intakes of breakfast cereals, low fat and skimmed milks, potatoes, fruit, fish, and fish dishes [16]
2-aminoadipate	A healthy cluster: higher intakes of breakfast cereals, low fat and skimmed milks, potatoes, fruit, fish, and fish dishes [16]
Glycylproline	An unhealthy cluster: higher intakes of chips/processed potatoes, meat products, savory snacks, and high-energy beverages [7]
N-aceytalglutamate	An unhealthy cluster: higher intakes of chips/processed potatoes, meat products, savory snacks, and high-energy beverages [7]
Theophylline	An unhealthy cluster: higher intakes of chips/processed potatoes, meat products, savory snacks, and high-energy beverages [7]

Reference

1. Stella, C.; Beckwith-Hall, B.; Cloarec, O.; Holmes, E.; Lindon, J.C.; Powell, J.; van der Ouderaa, F.; Bingham, S.; Cross, A.J.; Nicholson, J.K. Susceptibility of human metabolic phenotypes to dietary modulation. *J. Proteome Res.* **2006**, *5*, 2780–2788.
2. Xu, J.; Yang, S.; Cai, S.; Dong, J.; Li, X.; Chen, Z. Identification of biochemical changes in lactovegetarian urine using 1H NMR spectroscopy and pattern recognition. *Anal. Bioanal. Chem.* **2010**, *396*, 1451–1463.

3 Andersen, M.-B.S.; Kristensen, M.; Manach, C.; Pujos-Guillot, E.; Poulsen, S.K.; Larsen, T.M.; Astrup, A.; Dragsted, L. Discovery and validation of urinary exposure markers for different plant foods by untargeted metabolomics. Anal. Bioanal. Chem. 2014, 406, 1829–1844.

4 Barton, S.; Navarro, S.L.; Buas, M.F.; Schwarz, Y.; Gu, H.; Djukovic, D.; Raftery, D.; Kratz, M.; Neuhouser, M.L.; Lampe, J.W. Targeted plasma metabolome response to variations in dietary glycemic load in a randomized, controlled, crossover feeding trial in healthy adults. Food Funct. 2015, 6, 2949–2956.

5 Zheng, H.; Lorenzen, J.K.; Astrup, A.; Larsen, L.H.; Yde, C.C.; Clausen, M.R.; Bertram, H.C. Metabolic Effects of a 24-Week Energy-Restricted Intervention Combined with Low or High Dairy Intake in Overweight Women: An NMR-Based Metabolomics Investigation. Nutrients 2016, 8, 108.

6 O'Sullivan, A.; Gibney, M.J.; Brennan, L. Dietary intake patterns are reflected in metabolomic profiles: Potential role in dietary assessment studies. *Am. J. Clin. Nutr.* **2011**, *93*, 314–321.

7 Esko, T.; Hirschhorn, J.N.; Feldman, H.A.; Hsu, Y.-H.H.; Deik, A.A.; Clish, C.B.; Ebbeling, C.B.; Ludwig, D.S. Metabolomic profiles as reliable biomarkers of dietary composition. *Am. J. Clin. Nutr.* **2017**, *105*, 547–554.

8 Vázquez-Fresno, R.; Llorach, R.; Urpi-Sarda, M.; Lupianez-Barbero, A.; Estruch, R.; Corella, D.; Fitó, M.; Arós, F.; Ruiz-Canela, M.; Salas-Salvadó, J.; et al. Metabolomic Pattern Analysis after Mediterranean Diet Intervention in a Nondiabetic Population: A 1- and 3-Year Follow-up in the PREDIMED Study. J. Proteome Res. 2015, 14, 531–540.

9 Garcia-Perez, I.; Posma, J.M.; Gibson, R.; Chambers, E.S.; Hansen, T.H.; Vestergaard, H.; Hansen, T.; Beckmann, M.; Pedersen, O.; Elliott, P.; et al. Objective assessment of dietary patterns by use of metabolic phenotyping: A randomised, controlled, crossover trial. Lancet Diabetes Endocrinol. 2017, 5, 184–195.

10 Johansson-Persson, A.; Barri, T.; Ulmius, M.; Onning, G.; Dragsted, L.O. LC-QTOF/MS metabolomic profiles in human plasma after a 5-week high dietary fiber intake. Anal. Bioanal. Chem. 2013, 405, 4799–4809.

11 Rasmussen, L.G.; Winning, H.; Savorani, F.; Ritz, C.; Engelsen, S.B.; Astrup, A.; Larsen, T.M.; Dragsted, L.O. Assessment of dietary exposure related to dietary GI and fibre intake in a nutritional metabolomic study of human urine. Genes Nutr. 2012, 7, 281–293.

12 Van Dorsten, F.A.; Daykin, C.A.; Mulder, T.P.J.; Van Duynhoven, J.P.M. Metabonomics approach to determine metabolic differences between green tea and black tea consumption. J. Agric. Food Chem. 2006, 54, 6929–6938.

13 Law, W.S.; Huang, P.Y.; Ong, E.S.; Ong, C.N.; Li, S.F.Y.; Pasikanti, K.K.; Chan, E.C.Y. Metabonomics investigation of human urine after ingestion of green tea with gas chromatography/mass spectrometry, liquid chromatography/mass spectrometry and (1)H NMR spectroscopy. Rapid Commun. Mass Spectrom. RCM 2008, 22, 2436–2446.

14 May, D.H.; Navarro, S.L.; Ruczinski, I.; Hogan, J.; Ogata, Y.; Schwarz, Y.; Levy, L.; Holzman, T.; McIntosh, M.W.; Lampe, J.W. Metabolomic profiling of urine: Response to a randomised, controlled feeding study of select fruits and vegetables, and application to an observational study. Br. J. Nutr. 2013, 110, 1760–1770.

15 Hanhineva, K.; Lankinen, M.A.; Pedret, A.; Schwab, U.; Kolehmainen, M.; Paananen, J.; de Mello, V.; Sola, R.; Lehtonen, M.; Poutanen, K.; et al. Nontargeted Metabolite Profiling Discriminates Diet-Specific Biomarkers for Consumption of Whole Grains, Fatty Fish, and Bilberries in a Randomized Controlled Trial. J. Nutr. 2015, 145, 7–17.

16 Gibbons, H.; Carr, E.; McNulty, B.A.; Nugent, A.P.; Walton, J.; Flynn, A.; Gibney, M.J.; Brennan, L. Metabolomic-based identification of clusters that reflect dietary patterns. *Mol. Nutr. Food Res.* **2017**, *61*, 1601050.

17 Menni, C.; Zhai, G.; MacGregor, A.; Prehn, C.; Römisch-Margl, W.; Suhre, K.; Adamski, J.; Cassidy, A.; Illig, T.; Spector, T.D.; et al. Targeted metabolomics profiles are strongly correlated with nutritional patterns in women. Metabolomics 2013, 9, 506–514.

18 Floegel, A.; von Ruesten, A.; Drogan, D.; Schulze, M.B.; Prehn, C.; Adamski, J.; Pischon, T.; Boeing, H. Variation of serum metabolites related to habitual diet: A targeted metabolomic approach in EPIC-Potsdam. Eur. J. Clin. Nutr. 2013, 67, 1100–1108.

19 Schmidt, J.A.; Rinaldi, S.; Ferrari, P.; Carayol, M.; Achaintre, D.; Scalbert, A.; Cross, A.J.; Gunter, M.J.; Fensom, G.K.; Appleby, P.N.; et al. Metabolic profiles of male meat eaters, fish eaters, vegetarians, and vegans from the EPIC-Oxford cohort. Am. J. Clin. Nutr. 2015, 102, 1518–1526.

20 Stanstrup, J.; Schou, S.S.; Holmer-Jensen, J.; Hermansen, K.; Dragsted, L.O. Whey protein delays gastric emptying and suppresses plasma fatty acids and their metabolites compared to casein, gluten, and fish protein. J. Proteome Res. 2014, 13, 2396–2408.

21 Jacobs, S.; Kröger, J.; Floegel, A.; Boeing, H.; Drogan, D.; Pischon, T.; Fritsche, A.; Prehn, C.; Adamski, J.; Isermann, B.; et al. Evaluation of various biomarkers as potential mediators of the association between coffee consumption and incident type 2 diabetes in the EPIC-Potsdam Study. Am. J. Clin. Nutr. 2014, 100, 891–900.

22 Bouchard-Mercier, A.; Rudkowska, I.; Lemieux, S.; Couture, P.; Vohl, M.-C. The metabolic signature associated with the Western dietary pattern: A cross-sectional study. Nutr. J. 2013, 12, 158.

23 Garcia Aloy, M.; Llorach, R.; Urpí Sardà, M.; Jáuregui Pallarés, O.; Corella Piquer, D.; Ruiz-Canela, M.; Salas Salvadó, J.; Fitó Colomer, M.; Ros Rahola, E.; Riba, R.E.; et al. A metabolomics-driven approach to predict cocoa product consumption by designing a multimetabolite biomarker model in free-living subjects from the PREDIMED study. Mol. Nutr. Food Res. 2015, 59, 212–220.

24 Edmands, W.M.; Ferrari, P.; Rothwell, J.A.; Rinaldi, S.; Slimani, N.; Barupal, D.K.; Biessy, C.; Jenab, M.; Clavel-Chapelon, F.; Fagherazzi, G.; et al. Polyphenol metabolome in human urine and its association with intake of polyphenol-rich foods across European countries. Am. J. Clin. Nutr. 2015, 102, 905–913.

25 Clinical phenotype clustering in cardiovascular risk patients for the identification of responsive metabotypes after red wine polyphenol intake. J. Nutr. Biochem. 2016, 28, 114–120.

26 Llorach, R.; Garrido, I.; Monagas, M.; Urpi-Sarda, M.; Tulipani, S.; Bartolome, B.; Andres-Lacueva, C. Metabolomics study of human urinary metabolome modifications after intake of almond (Prunus dulcis (Mill.) D.A. Webb) skin polyphenols. J. Proteome Res. 2010, 9, 5859–5867.

27 Rangel-Huerta, O.D.; Aguilera, C.M.; Perez-de-la-Cruz, A.; Vallejo, F.; Tomas-Barberan, F.; Gil, A.; Mesa, M.D. A serum metabolomics-driven approach predicts orange juice consumption and its impact on oxidative stress and inflammation in subjects from the BIONAOS study. Mol. Nutr. Food Res. 2017, 61, 1600120.

28 Heinzmann, S.S.; Brown, I.J.; Chan, Q.; Bictash, M.; Dumas, M.-E.; Kochhar, S.; Stamler, J.; Holmes, E.; Elliott, P.; Nicholson, J.K. Metabolic profiling strategy for discovery of nutritional biomarkers: Proline betaine as a marker of citrus consumption. *Am. J. Clin. Nutr.* **2010**, *92*, 436–443.

29 Pujos-Guillot, E.; Hubert, J.; Martin, J.-F.; Lyan, B.; Quintana, M.; Claude, S.; Chabanas, B.; Rothwell, J.A.; Bennetau-Pelissero, C.; Scalbert, A.; et al. Mass spectrometry-based metabolomics for the discovery of biomarkers of fruit and vegetable intake: Citrus fruit as a case study. J. Proteome Res. 2013, 12, 1645–1659.

30 Edmands, W.M.B.; Beckonert, O.P.; Stella, C.; Campbell, A.; Lake, B.G.; Lindon, J.C.; Holmes, E.; Gooderham, N.J. Identification of human urinary biomarkers of cruciferous vegetable consumption by metabonomic profiling. *J. Proteome Res.* **2011**, *10*, 4513–4521.

31 Bondia-Pons, I.; Cañellas, N.; Abete, I.; Rodríguez, M.Á.; Perez-Cornago, A.; Navas-Carretero, S.; Zulet, M.Á.; Correig, X.; Martínez, J.A. Nutri-metabolomics: Subtle serum metabolic differences in healthy subjects by NMR-based metabolomics after a short-term nutritional intervention with two tomato sauces. Omics J. Integr. Biol. 2013, 17, 611–618.

32 Moazzami, A.A.; Shrestha, A.; Morrison, D.A.; Poutanen, K.; Mykkänen, H. Metabolomics reveals differences in postprandial responses to breads and fasting metabolic characteristics associated with postprandial insulin demand in postmenopausal women. J. Nutr. 2014, 144, 807–814.

33 Altmaier, E.; Kastenmüller, G.; Römisch-Margl, W.; Thorand, B.; Weinberger, K.M.; Adamski, J.; Illig, T.; Döring, A.; Suhre, K. Variation in the human lipidome associated with coffee consumption as revealed by quantitative targeted metabolomics. Mol. Nutr. Food Res. 2009, 53, 1357–1365.

34 Altmaier, E.; Kastenmüller, G.; Römisch-Margl, W.; Thorand, B.; Weinberger, K.M.; Illig, T.; Adamski, J.; Döring, A.; Suhre, K. Questionnaire-based self-reported nutrition habits associate with serum metabolism as revealed by quantitative targeted metabolomics. Eur. J. Epidemiol. 2011, 26, 145–156.

35 Bondia-Pons, I.; Martinez, J.A.; de la Iglesia, R.; Lopez-Legarrea, P.; Poutanen, K.; Hanhineva, K.; Zulet, M. de los Á. Effects of short- and long-term Mediterranean-based dietary treatment on plasma LC-QTOF/MS metabolic profiling of subjects with metabolic syndrome features: The Metabolic Syndrome Reduction in Navarra (RESMENA) randomized controlled trial. *Mol. Nutr. Food Res.* **2015**, *59*, 711–728.

36 Stalmach, A.; Mullen, W.; Barron, D.; Uchida, K.; Yokota, T.; Cavin, C.; Steiling, H.; Williamson, G.; Crozier, A. Metabolite profiling of hydroxycinnamate derivatives in plasma and urine after the ingestion of coffee by humans: Identification of biomarkers of coffee consumption. Drug Metab. Dispos. Biol. Fate Chem. 2009, 37, 1749–1758.

37 Redeuil, K.; Smarrito-Menozzi, C.; Guy, P.; Rezzi, S.; Dionisi, F.; Williamson, G.; Nagy, K.; Renouf, M. Identification of novel circulating coffee metabolites in human plasma by liquid chromatography-mass spectrometry. J. Chromatogr. A 2011, 1218, 4678–4688.

38 First identification of dimethoxycinnamic acids in human plasma after coffee intake by liquid chromatography-mass spectrometry. J. Chromatogr. A 2011, 1218, 491–497.

39 Schröder, H.; de la Torre, R.; Estruch, R.; Corella, D.; Martínez-González, M.A.; Salas-Salvadó, J.; Ros, E.; Arós, F.; Flores, G.; Civit, E.; et al. Alcohol consumption is associated with high concentrations of urinary hydroxytyrosol. Am. J. Clin. Nutr. 2009, 90, 1329–1335.

40 Perez-Jimenez, F.; Alvarez de Cienfuegos, G.; Badimon, L.; Barja, G.; Battino, M.; Blanco, A.; Bonanome, A.; Colomer, R.; Corella-Piquer, D.; Covas, I.; et al. International conference on the healthy effect of virgin olive oil. Eur. J. Clin. Investig. 2005, 35, 421–424.

41 Bondia-Pons, I.; Barri, T.; Hanhineva, K.; Juntunen, K.; Dragsted, L.O.; Mykkänen, H.; Poutanen, K. UPLC-QTOF/MS metabolic profiling unveils urinary changes in humans after a whole grain rye versus refined wheat bread intervention. Mol. Nutr. Food Res. 2013, 57, 412–422.

42 Nestel, P.J.; Straznicky, N.; Mellett, N.A.; Wong, G.; De Souza, D.P.; Tull, D.L.; Barlow, C.K.; Grima, M.T.; Meikle, P.J. Specific plasma lipid classes and phospholipid fatty acids indicative of dairy food consumption associate with insulin sensitivity. Am. J. Clin. Nutr. 2014, 99, 46–53.
